# Anti-Tumor Potency of Short-Term Interleukin-15 Dendritic Cells Is Potentiated by *In Situ* Silencing of Programmed-Death Ligands

**DOI:** 10.3389/fimmu.2022.734256

**Published:** 2022-02-17

**Authors:** Maarten Versteven, Donovan Flumens, Diana Campillo-Davó, Hans De Reu, Laura Van Bruggen, Stefanie Peeters, Viggo Van Tendeloo, Zwi Berneman, Harry Dolstra, Sébastien Anguille, Willemijn Hobo, Evelien Smits, Eva Lion

**Affiliations:** ^1^ Laboratory of Experimental Hematology, Vaccine and Infectious Disease Institute (VAXINFECTIO), Faculty of Medicine and Health Sciences, University of Antwerp, Antwerp, Belgium; ^2^ Division of Hematology, Antwerp University Hospital, Edegem, Belgium; ^3^ Center for Cell Therapy and Regenerative Medicine, Antwerp University Hospital, Edegem, Belgium; ^4^ Department of Laboratory Medicine – Laboratory of Hematology, Radboud University Medical Center, Nijmegen, Netherlands; ^5^ Center for Oncological Research (CORE), Faculty of Medicine and Health Sciences, University of Antwerp, Antwerp, Belgium

**Keywords:** dendritic cells, interleukin-15, programmed death (PD)-1, programmed death 1 ligand, siRNA, Wilms’ tumor 1 (WT1)

## Abstract

Dendritic cell (DC) vaccines have proven to be a valuable tool in cancer immune therapy. With several DC vaccines being currently tested in clinical trials, knowledge about their therapeutic value has been significantly increased in the past decade. Despite their established safety, it has become clear that objective clinical responses are not yet robust enough, requiring further optimization. Improvements of this advanced therapy medicinal product encompass, among others, regulating their immune stimulating capacity by *in situ* gene engineering, in addition to their implementation in combination therapy regimens. Previously, we have reported on a superior monocyte-derived DC preparation, including interleukin-15, pro-inflammatory cytokines and immunological danger signals in the culture process. These so-called IL-15 DCs have already proven to exhibit several favorable properties as cancer vaccine. Evolving research into mechanisms that could further modulate the immune response towards cancer, points to programmed death-1 as an important player that dampens anti-tumor immunity. Aiming at leveraging the immunogenicity of DC vaccines, we hypothesized that additional implementation of the inhibitory immune checkpoint molecules programmed death-ligand (PD-L)1 and PD-L2 in IL-15 DC vaccines would exhibit superior stimulatory potential. In this paper, we successfully implemented PD-L silencing at the monocyte stage in the 3-day IL-15 DC culture protocol resulting in substantial downregulation of both PD-L1 and PD-L2 to levels below 30%. Additionally, we validated that these DCs retain their specific characteristics, both at the level of phenotype and interferon gamma secretion. Evaluating their functional characteristics, we demonstrate that PD-L silencing does not affect the capacity to induce allogeneic proliferation. Ultimately designed to induce a durable tumor antigen-specific immune response, PD-L silenced IL-15 DCs were capable of surpassing PD-1-mediated inhibition by antigen-specific T cells. Further corroborating the superior potency of short-term IL-15 DCs, the combination of immune stimulatory components during DC differentiation and maturation with *in situ* checkpoint inhibition supports further clinical translation.

## Introduction

Ever since the introduction of dendritic cells (DCs) in the field of cancer immunotherapy (1), extensive research has been done to exploit this therapeutic modality (2–5). From early clinical trials, it is now generally accepted that DC vaccination is well tolerated and safe (6). While DC vaccination can elicit immune responses in many patients, objective clinical responses remain limited and prone to improvement. Our group previously reported the development of a novel DC type, by differentiating monocytes with granulocyte-macrophage colony stimulating factor (GM-CSF) complemented with the pleiotropic cytokine interleukin (IL)-15—instead of the more classically used IL-4—and a maturation cocktail including interferon (IFN)-γ, prostaglandin E2 (PGE2), tumor necrosis factor (TNF)-α and a Toll-like receptor (TLR) 7/8 agonist (7). In the past decade, we extensively researched this type of monocyte-derived DC (moDC), hereafter referred to as IL-15 DC, demonstrating its superior immunostimulatory capacity. First, we showed that IL-15 DCs are superior in terms of their capacity to induce both T helper 1 and cytotoxic T-cell responses (7, 8) and to potentiate natural killer (NK) cell and gamma delta (γδ) T cell cytotoxicity (9–12). Moreover, IL-15 DCs have intrinsic cytotoxic properties, allowing them to be listed as ‘killer DCs’ (8, 13). Interestingly, IL-15 DCs are able to secrete the immune regulatory cytokines IFN-γ and IL-15 and granzyme B, which contribute to their direct cytotoxic efficacy (8).

In our previous clinical trials with conventional IL-4 DC vaccines [NCT01686334 and NCT00834002 for acute myeloid leukemia (AML)] we have observed favorable objective responses. More clinical research with these IL-4 DCs is currently being conducted by our group (NCT02649829 for mesothelioma, NCT02649582 for glioblastoma, NCT01291420 for solid tumors). IL-4 DC-vaccination prolonged relapse-free survival in AML patients; however, not all IL-4 DC-vaccinated patients responded equally well, and the majority of responders eventually relapsed (14). This disparity in responses seen in IL-4 DC-vaccinated patients and the suboptimal efficacy of the treatment can be partially explained by the presence of costimulatory and inhibitory signals, whose balance are involved in determining the strength of an immune response. The presence of inhibitory immune checkpoints and/or their ligands on the surface of DCs, has been demonstrated *in vivo* in mice to hamper their stimulatory capacity towards immune effector cells. Indeed, programmed death 1 (PD-1) ligand 1 (PD-L1) is highly expressed on DCs and blockade of PD-L1 can reactivate tumor-infiltrating T cells (15). Merging the evolving science on the role of PD-1/PD-L signaling in DC-mediated immunity and the pursuit of developing highly immunogenic DC vaccines, a new therapeutic approach to interfere with the PD-1/PD-L pathway has been introduced by Dolstra et al. They showed that conventional IL-4 DCs express high levels of the co-inhibitory molecules PD-L1 and PD-L2, which can be downregulated following transfection with specific short interfering or silencing RNA (siRNA) (16–18). These PD-L-silenced antigen-loaded DCs superiorly boosted *ex vivo* and *in vivo* minor histocompatibility antigen-specific T cell responses from leukemia patients (17). The safety and feasibility of these DCs to induce more robust clinical responses is currently being investigated (NCT02528682). Given this successful improvement of the stimulatory capacity of IL4 DCs, we subsequently showed that combining *in situ* downregulation of PD-L1 and PD-L2 with introduction of interleukin-15 transpresentation tools could further potentiate tumor-reactive T-cell expansion (19).

Extending the preclinical development of short-term IL-15 DCs as therapeutic cancer vaccine, the aim of this study was to confirm the added value of *in situ* PD-L1 and PD-L2 silencing of IL-15 DCs, harnessed with a unique immune-stimulating profile that significantly outperforms IL-4 DC-mediated *in vitro* anti-tumor activity (7–13). Following successful development and qualification of a PD-L silencing protocol for 3-day cultured IL-15 DCs, their functional capacity to regulate proliferation and immunostimulatory cytokine production by allogeneic and autologous primary lymphocytes was evaluated. Ultimately, by targeting the tumor-associated antigen Wilms’ tumor-1 (WT1), the tumor-antigen specificity was assessed by demonstrating a superior antigen-specific T cell stimulating capacity of PD-L-silenced IL-15 DCs. The data presented here provide a rationale for implementing PD-L silenced IL-15 DCs as next generation anticancer vaccines in upcoming clinical trials.

## Materials and Methods

### Ethics Statement and Primary Cell Material

This study was approved by the Ethics Committee of the University Hospital Antwerp/University of Antwerp (Antwerp, Belgium) under reference number 16/10/123. Peripheral blood mononuclear cells (PBMCs) were isolated from anonymous donor buffy coats provided by the Blood Service of the Flemish Red Cross (Mechelen, Belgium) by means of Ficoll density gradient centrifugation. Positively selected CD14^+^ monocytes were isolated from PBMCs by means of CD14^+^ magnetic microbeads for magnetic-activated cell sorting (MACS; Miltenyi Biotec, Leiden, The Netherlands) and were freshly cultured into IL-15DCs (*vide infra*). CD14^-^ peripheral blood lymphocytes (PBLs) were cryopreserved or used for isolation of NK cells by negative immunoselection using the NK cell isolation kit (Miltenyi Biotec). PBLs and NK cells were cryopreserved at a maximum cell concentration of 100x10^6^/mL in 1 mL fetal bovine serum (FBS, Life Technologies, Merelbeke, Belgium) supplemented with 10% dimethyl sulfoxide (DMSO, Sigma-Aldrich, Diegem, Belgium) per cryovial.

### Cell Lines

The HLA-A*02:01 positive, WT1-negative multiple myeloma cell line U266 was kindly provided by Prof. W. Germeraad (Maastricht University Medical Center, Maastricht, The Netherlands). T cell receptor (TCR)-deficient Jurkat J76.7 cells transduced to express eGFP after activation of a specific introduced TCR, hereafter called 2D3 cells, were kindly provided by prof. H. Sugiyama (Osaka University Graduate School of Medicine, Osaka, Japan) under material transfer agreement (MTA)19-308. PD-1^+^ 2D3 cells were stably transduced with PD-1 by prof. K. Breckpot (Free University Brussels, Brussels, Belgium) (20). All cell lines were maintained in Roswell Park Memorial Institute medium (RPMI; ThermoFisher Scientific) supplemented with 10% FBS.

### mRNAs and siRNAs

Codon-optimized Sig-DC-LAMP *WT1* mRNA encoding isoform D of WT1 (14) was used to transfect antigen-presenting cells. The human WT1_37-45_-specific TCR gene was generated as described in (21). The coding sequence of PD-1 was cloned into the pST1 [RHAMM] vector using SpeI and XhoI cloning sites to produce [pST1 PDCD1 vector] (GeneArt, ThermoFisher Scientific). The original pST1 backbone vector was kindly provided by Dr. Ugur Sahin (Johannes-Gutenberg University, Mainz, Germany) (22) under MTA. Plasmid DNA was propagated as described in (21). Next, plasmids were linearized after the 120 bp poly(A) tail using SapI restriction enzyme (ThermoFisher Scientific). mRNA was synthesized from linearized plasmid template using an mMessage mMachine T7 *in vitro* transcription kit (ThermoFisher Scientific) according to the manufacturer’s instructions. PD-L1 and PD-L2-targeting siRNAs as well as control luciferase-targeting siRNAs were produced as described in (17, 18) and were kindly delivered by Prof. H. Dolstra and Prof. W. Hobo (Radboud University medical center, Nijmegen, The Netherlands).

### Generation of IL-15 DCs

The culture protocol of short-term IL-15 DCs was adapted from the previously described protocol (7) to include the disruption of newly synthesized PD-L1 and PD-L2 (**Supplementary Figure S1**). Isolated CD14^+^ monocytes were transfected in GMP-ready serum-free phenol red-free X-VIVO15 medium (Lonza, Verviers, Belgium). For each transfection, optimized amounts of 2 µg siRNAs (luciferase (luci) or PD-L1/PD-L2 (PD-L), 2:1 ratio) were pre-incubated with 10 µL SAINT-RED (Synvolux, Leiden, The Netherlands) per mL transfection volume. After 15 minutes, 4.5–6.0 x 10^6^ monocytes were resuspended in X-VIVO15 per mL transfection volume and transferred to a T75 culture flask (7 mL transfection volume) or T175 culture flask (15 mL transfection volume). For the non-transfected condition (no), neither siRNAs nor SAINT-RED was used. After 1 hour of lipofection at 37°C, differentiation medium was added to a total volume of 21 mL in T75 or 45 mL in T175 flasks, resulting in a cell culture density of 1.5–2 x 10^6^ monocytes/mL. Differentiation medium was prepared for final culture medium (transfection volume + differentiation medium) concentrations of 800 IU/mL GM-CSF, 200 ng/mL IL-15 and 1% hAB serum (Life Technologies). After 48 h, a maturation cocktail containing 3 µg/mL TLR 7/8 ligand R-848 (Enzo Life Sciences, Antwerp, Belgium), 2.5 ng/mL TNF-α (Gentaur, Brussels, Belgium), 250 ng/mL IFN-γ (Immunotools, Friesoythe, Germany) and 1 µg/mL PGE2 (Prostin E2, Pfizer, Puurs, Belgium) was added for 16-20 hours (12). Mature PD-L-silenced IL-15 DCs were harvested and cryopreserved at 5-15 x 10^6^ cells per vial in 70% X-VIVO15 medium, 20% hAB serum (Life Technologies) and 10% DMSO.

### Membrane Phenotyping of IL-15 DCs

For membrane immunophenotyping of IL-15 DCs, FcRγIII receptors on IL-15 DCs were blocked using mouse gamma-globulins (Jackson Immunoresearch, Suffolk, UK). IL-15 DCs were characterized by immunofluorescent surface staining using fluorescein isothiocyanate (FITC)-conjugated monoclonal antibodies (mABs) recognizing CD83, CD274 (PD-L1) and IL-15, phycoerythrin (PE)-conjugated mABs recognizing CD14, CD56, CD80 and CD273 (PD-L2) or PE-Cy7-conjugated mABs recognizing CD7 and CD86 (BD Biosciences, Erembodegem, Belgium; Invitrogen, Camarillo, CA, USA; R&D Systems, Minneapolis, MN, USA). Corresponding species- and isotype-matched antibodies were used as negative controls. Viability was determined using either propidium iodine (PI; Invitrogen) or 7-aminoactinomycin D (7-AAD; BD Biosciences). Samples were acquired on a CytoFLEX flow cytometer (Beckman Coulter, Suarlée, Belgium).

### Electroporation

Cells were electroporated in a 4-mm cuvette (Cell Projects, Harrietsham, UK) in a Gene Pulser Xcell electroporator (Biorad, Temse, Belgium) using 1 µg mRNA per 10^6^ cells. Fresh 5-10 x 10^6^ IL-15 DCs were electroporated with *WT1* mRNA in 250 µL OptiMEM (ThermoFisher Scientific) with an exponential decay pulse (300V, 150 µF) (7). U266, used as control antigen presenting cells (APCs), were treated identically but electroporated using a time constant pulse (300V, 8 ms) (23). *WT1* mRNA transfection efficiency was determined 4 hours post electroporation by means of intracellular staining employing the eBioscience FoxP3/transcription factor intracellular staining buffer set (Invitrogen) and an anti-WT1 primary antibody (clone 6F-H2, Dako, Agilent, CA, US) as described previously (23). 10-20 x 10^6^ PD-1^+^ 2D3 cells were electroporated in 400 µL OptiMEM with *TCR* mRNA as described before (20). 20-40 x 10^6^ thawed PBLs and purified NK cells were electroporated in 500 µL OptiMEM with *PD-1* mRNA using a square wave protocol (500V, 5 ms). TCR and PD-1 protein expression was evaluated 2 hours post electroporation by surface staining with pan-TCRαβ-PE (Miltenyi Biotec) and CD279-FITC (BD Biosciences), respectively. All acquisitions were performed on a CytoFLEX flow cytometer.

### Peptide-Loading of IL-15 DCs

Alternatively to electroporating full-length *WT1* mRNA, IL-15 DCs were peptide-pulsed with WT1_37-45_ peptide (VLDFAPPGA; JPT, Berlin, Germany). DCs were resuspended in serum-free RPMI medium at a concentration of 2 x 10^6^ IL-15 DCs/mL in polypropylene tubes. 10 µg/mL of the peptide was added, and cells were incubated at room temperature on a horizontal tilting tube roller covered from direct light. After 1 hour, cells were washed and resuspended in RPMI supplemented with 10% FBS for use in functional assays.

### Allogeneic Mixed Lymphocyte Reaction (Allo-MLR)

PBL from an allogeneic blood donor were thawed, transfected with *PD-1* mRNA (*vide supra*) or mock transfected (no mRNA), and stained with 5,6-carboxyfluorescein diacetate succinimidyl ester (CFSE; 5 µM, Invitrogen) as previously described (8), prior to co-culture with IL-15 DCs at an APC : PBL ratio of 1:10. Stimulation with phytohemagglutinin (PHA; 1µg/mL; Sigma-Aldrich, Overijse, Belgium) and IL-2 (20 IU/mL; Immunotools) served as a positive control (8). After 5 days, co-culture supernatant was collected and stored at -20°C until further use (ELISA, *vide infra*). Cell pellets were washed and stained with CD3-PerCP-Cy5.5, CD4-APC, CD8-Brilliant Violet 786, CD56-PE and CD279-Brilliant Violet 421 (all from BD Biosciences). Fixable Aqua dead cell stain (ThermoFisher Scientific) was used to discriminate between viable and dead cells. Samples were acquired on a FACSAria II flow cytometer (BD Biosciences). An example of the applied gating strategy is depicted in **Supplementary Figure 2**.

### DC-Mediated NK Cell Activity Assay

To evaluate the NK cell-stimulating capacity of the PD-L-disrupted IL-15 DCs, DCs were co-cultured with autologous non-electroporated or *PD-1* mRNA-electroporated purified NK cells in 96-well round-bottom plates at a ratio of 1:1 (250.000 cells per cell type) in triplicate at 37°C. 24h and 48h cell-free culture supernatant was collected and stored at -20°C for assessment of NK cell-mediated IFN-γ secretion (*vide infra*).

### WT1-Specific PD-1^+^ 2D3 T Cell Assay

WT1_37-45_ specific *TCR* mRNA-transfected PD-1^+^ 2D3 cells (20) were co-cultured in 96-well plates with no/luci/PDL siRNA IL-15 DCs or U266 cells (as positive control) that were electroporated with *WT1* mRNA or peptide-pulsed with WT1_37-45_ peptide or left unpulsed at a 2D3:APC ratio of 2:1 (100.000:50.000 cells per well). When indicated, 1x10^6^/mL 2D3 cells were pre-incubated for 1 hour with 15µg/mL anti-PD-1 antibody nivolumab (kindly provided by the pharmacy of the Antwerp University Hospital), prior to co-culture with APCs. After 16-hour co-culture at 37°C, supernatant was collected and stored at -20°C until further use. Cell pellets were stained with CD8-PE, washed, incubated for 10 minutes with 7-AAD and subsequently assessed for eGFP expression. Samples were acquired on a CytoFLEX flow cytometer. An example of the applied gating strategy is depicted in **Supplementary Figure 3**.

### ELISA

Secretion of IFN-γ by IL-15 DCs or lymphocytes was determined in 24-hour washout supernatant or 5-day co-culture supernatant, respectively), using an enzyme-linked immunosorbent assay (ELISA; Peprotech, US) following the manufacturer’s instructions. Samples were diluted when necessary to fit the standard curve of the kit with a detection limit of 16 pg/mL and top standard of 2000 pg/mL. Secretion of granzyme B by 2D3 cells and IL-15 DCs was determined in 16-hour co-culture supernatant or 48h monoculture washout supernatant using ELISA (R&D systems) following the manufacturer’s instructions. A sample dilution of 1:8 was optimal to fit the standard curve of the kit (with a detection limit of 24.4 pg/mL and top standard of 2500 pg/mL) when analyzing the 2D3 coculture supernatant, while supernatant from monocultures was measured undiluted. For both ELISAs immunoluminescence was measured on a Victor 3 multilabel counter (Perkin Elmer).

### Statistical Analysis

Data were statistically analyzed and represented with GraphPad Prism (version 9, CA, US). The data was checked for normal distribution and homogeneous variances. When data were normally distributed parametric analyses were performed. When normal distribution could not be confirmed, non-parametric analyses were performed. Data are expressed as mean ± standard error of mean (SEM). *P*-values smaller than 0.05 were considered statistically significant (*p ≤ 0.05; **p ≤ 0.01; ***p ≤ 0.001).

## Results

### Highly Efficient *In Situ* PD-L1/2 Silencing in Short-Term IL-15 DCs

**Figure 1:**
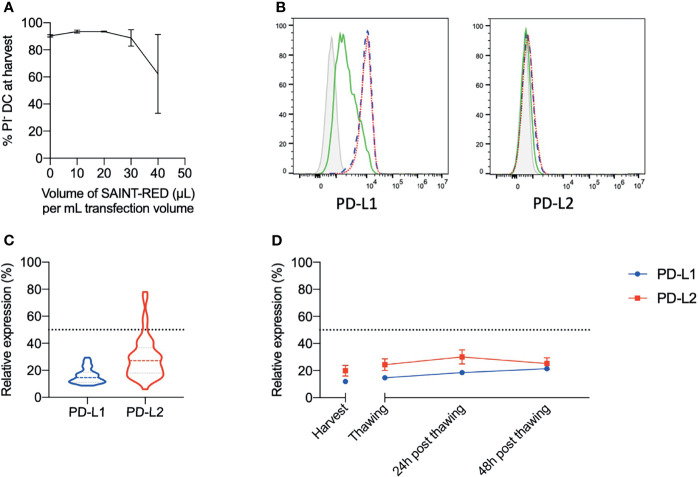
Optimization of siRNA transfection protocol in monocytes and assessment of transfection efficiency assessed on mature IL-15 DCs. **(A)** The effect of increasing concentrations of SAINT-RED lipofectant (expressed in µL/mL transfection volume for 4.5-6.0 x 10^6^ monocytes/mL) on mature IL-15 DC viability at harvest was assessed flow cytometrically with PI (n = 3). **(B)** Representative histogram overlays of the surface expression of PD-1 ligands PD-L1 and PD-L2 in mature IL-15 DCs at harvest from untreated (blue dashed line), luciferase siRNA-treated (red dotted line), and PD-L siRNA-treated (green line) monocytes. Grey filled line represents the corresponding isotypes. **(C)** Relative expression of PD-L1 and PD-L2 in mature PD-L siRNA IL-15 DCs compared to luci siRNA IL-15 DCs of PD-L1 and PD-L2 at harvest (n = 48). The horizontal dotted line indicates a cut-off value of 50% relative expression as indicator of successful silencing. **(D)** Kinetics of surface PD-L1 and PD-L2 relative expression on fresh mature IL-15 DCs at harvest and 1, 24 or 48 hours after thawing (n = 8). PD-L1, programmed death-1 ligand 1; PD-L2, programmed death-1 ligand 2.

Our group previously reported a protocol for silencing monocyte-derived IL-4 DCs before maturation, in which 40 μL of SAINT-RED per mL were used for transfecting siRNAs (19). However, we observed that this concentration of SAINT-RED resulted in low IL-15 DC viability with a very high experimental variability between replicates (62.3 ± 29.10 % PI^-^ IL-15 DC; **Figure 1A**). Therefore, to determine non-cytotoxic concentrations of lipofectant for transfection of purified monocytes prior to differentiation into IL-15 DCs, we performed a titration of SAINT-RED and assessed the viability of mature IL-15 DCs at harvest (**Figure 1A**). Monocytes treated with 20 µL or 10 µL/mL SAINT-RED, could be robustly differentiated into highly viable mature IL-15 DCs (93.50 ± 0.40% PI^-^ IL-15 DC and 93.50 ± 1.10% PI^-^ IL-15 DC, respectively) similar to their non-transfected counterparts (90.25 ± 0.85% PI^-^ IL-15 DC), whereas 30 µL/mL SAINT-RED seemed to have a cytotoxic effect at least in some donors (88.75 ± 6.15% PI^-^ IL-15 DC), albeit lower than 40 µL/mL. Continuing with10 µL SAINT-RED per mL transfection volume, the ratio of siRNA to SAINT-RED was further optimized. The ratio of 2:1 PD-L1:PD-L2 siRNA was kept as described previously (19), but total amount of siRNA was further downscaled to 2 µg/mL transfection volume due to the toxic effect of high concentrations of siRNA (data not shown). Silencing efficiency of PD-L siRNA lipofection was evaluated by calculating the relative expression of PD-L1 and PD-L2 on mature IL-15 DCs of the PD-L siRNA condition in relation to the paired control luciferase siRNA condition (ΔMFI PD-L condition/ΔMFI luci condition, with ΔMFI = MFI of PD-L expression – MFI of matched isotype). Similar ΔMFI of PD-L1 surface expression were observed on non-lipofected IL-15 DCs and on control luci siRNA IL-15 DCs (ΔMFI_PD-L1_ 8057 ± 515.9 and 8495 ± 603.6 (n=48), respectively; **Figure 1B**, left panel). Equally, IL-15 DC PD-L2 surface expression on untreated IL-15 DCs was similar to that of IL-15 DCs treated with control luci siRNA (ΔMFI_PD-L2_ 908.2 ± 306.2 and 1159 ± 392.1, respectively; **Figure 1B**, right panel) (n=48). Surface expression of both ligands was efficiently disrupted in mature siRNA IL-15 DCs, with a ΔMFI_PD-L1_ of 1288 ± 84.8 (n=48; *p* < 0.0001 compared to no siRNA and luci siRNA IL-15 DCs) and ΔMFI_PD-L2_ of 194.0 ± 58.7 (n=48, *p* = 0.0114 compared to no siRNA and luci siRNA IL-15 DCs). These data are summarized in **Table 1**. Successful silencing was set at a threshold value of 50% reduction in relative expression of PD-L1 and PD-L2 (**Figures 1C, D**). As shown in **Figure 1C**, calculated relative expression levels were far below the set threshold of 50% silencing, with a relative expression of 15.94 ± 0.80% for PD-L1 and 29.47 ± 2.40% for PD-L2. Stable PD-L1 and PD-L2 siRNA-mediated silencing was confirmed after thawing matured IL-15 DCs (14.71 ± 0.66% and 24.36 ± 4.38% relative expression of PD-L1 and PD-L2, respectively) and at different time points after thawing, as demonstrated by the constant low relative expression below the 50% cut-off value (**Figure 1D**).

**Table 1:** Summary of ΔMFI (MFI_PD-L_ – MFI_isotype_) of no siRNA IL-15 DCs (no), luciferase siRNA IL-15 DCs (luci) or PD-L siRNA IL-15 DCs (PD-L), n = 48.

	No	Luci	PD-L
PD-L1	8057 ± 515.9	8495 ± 603.6	1288 ± 84.8
PD-L2	908.2 ± 306.2	1159 ± 392.1	194 ± 58.7

### PD-L Silencing Has No Negative Impact on Mature IL-15 DCs Characteristics

**Figure 2:**
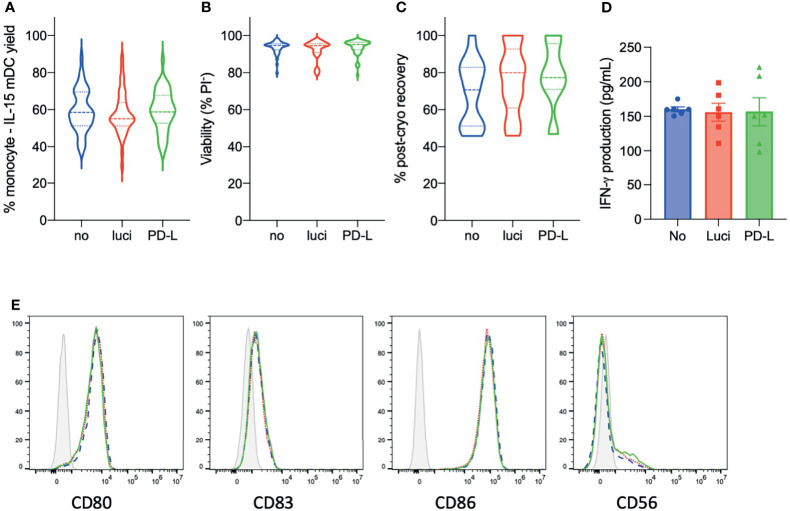
Phenotypic characterization of PD-L silenced mature IL15 DCs. Violin plots of **(A)** monocyte-to-mature IL-15 DC yields (n = 44), **(B)** viability (% PI^-^; n = 44) and **(C)** post-cryopreservation (post-cryo) recovery 15 to 30 min after thawing (n = 13) from untreated (no), control siRNA-treated (luci) and PD-L1/PD-L2 siRNA-treated (PD-L) IL-15 DCs at harvest. **(D)** Concentration of IFN-γ, an IL-15 DC hallmark, in 24-hour cell-free supernatants of IL-15 DC monocultures (100,000 cells; n = 6). **(E)** Representative histogram overlays of key surface markers of mature IL-15 DCs on no siRNA IL-15 DCs (blue dashed line) and PD-L siRNA IL-15 DCs (line histogram), including the corresponding isotype control (grey filled line). IFN-γ, interferon gamma; mDC, mature dendritic cells.

Confirming the reproducibility of the optimized PD-L siRNA IL-15 DC culture protocol, comparable and robust monocyte-to-mature IL-15 DC yields were obtained for untreated (58.02 ± 1.79%), luci siRNA-treated (56.68 ± 1.78%), and PD-L siRNA-treated (59.90 ± 1.59%) IL-15 DCs at harvest (n = 40; **Figure 2A** and **Table 1**). Viability of mature IL-15 DCs at harvest was maximal for all conditions (93.66 ± 0.49%, 92.03 ± 0.81% and 93.47 ± 0.62% PI^-^ for untreated, luci siRNA and PD-L siRNA IL-15 DCs, respectively; **Figure 2B** and **Table 2**). To anticipate the effect of siRNA-mediated silencing on reconstitution of thawed IL-15 DCs for vaccine administration, the pre-to-post-cryopreservation recovery was assessed (**Figure 2C**). While only 70.64 ± 5.57% of untreated IL-15 DCs could be viably recovered after cryopreservation, better post-cryopreservation recoveries were achieved for luci siRNA (81.45 ± 7.69%) and PD-L siRNA IL-15 DCs (83.15 ± 7.07%). Phenotypically, expression levels of conventional DC maturation markers CD80, CD83 and CD86, and the prototypic IL-15 DC surface marker CD56, did not differ between the untreated and siRNA-treated IL-15 DCs (**Figure 2E**). In line with this, secretion levels of the immune-regulator IFN-γ, a key feature of IL-15 DCs, were comparable among untreated IL-15 DCs (163.4 ± 6.19 pg/mL), control luci siRNA IL-15 DCs (164.7 ± 18.52 pg/mL) and PD-L siRNA IL-15 DCs (152.8 ± 31.69 pg/mL, n = 6) in 24-hour washout supernatants (**Figure 2D**). Furthermore, granzyme B secretion in 48h washout supernatant was studied as it is a hallmark of IL-15 DCs. While untreated and luci siRNA treated IL-15 DCs secreted 110.05 ± 17.58 pg/mL and 88.97 ± 8.35 pg/mL of granzyme B, respectively, PD-L siRNA treated IL-15 DCs secreted 75.98 ± 9.69 pg/mL (n=3). This difference was not significant, demonstrating retainment of the prototypic DC function. In summary, these data demonstrate that PD-L siRNA/SAINT-RED lipofection at the monocyte level is highly reproducible and has no impact on the yield and the prototypic characteristics of mature IL-15 DCs.

**Table 2:** Summary of yield, viability and post-cryopreservation (cryo) recovery of no siRNA IL-15 DCs (no), luciferase siRNA IL-15 DCs (luci) and PD-L siRNA IL-15 DCs (PD-L) cultured in 6-well plates or T75 flasks. Values are expressed as mean ± SEM.

		No	Luci	PD-L
Monocyte – IL-15 DCs yield	6-well plate	44.55 ± 3.75%	45.19 ± 2.42%	62.50 ± 2.42%
	T75	59.37 ± 1.81%	57.83 ± 1.85%	59.65 ± 1.73%
	All	58.02 ± 1.79%	56.68 ± 1.77%	59.90 ± 1.59%
Viability	6-well plate	94.38 ± 0.57%	86.83 ± 3.95%	91.68 ± 2.79%
	T75	93.59 ± 0.54%	92.51 ± 0.79%	93.64 ± 0.64%
	All	93.66 ± 0.49%	92.03 ± 0.81%	93.47 ± 0.62%
Post-cryo recovery		70.64 ± 5.58%	81.45 ± 7.69%	83.15 ± 7.07%

### *In Situ* PD-L Silencing Improves IL-15 DC-Mediated Primary Cell Allo-Stimulatory Capacity in the NK Cell Compartment, but Not Autologous PD-1^+^ NK Cell Activity

**Figure 3:**
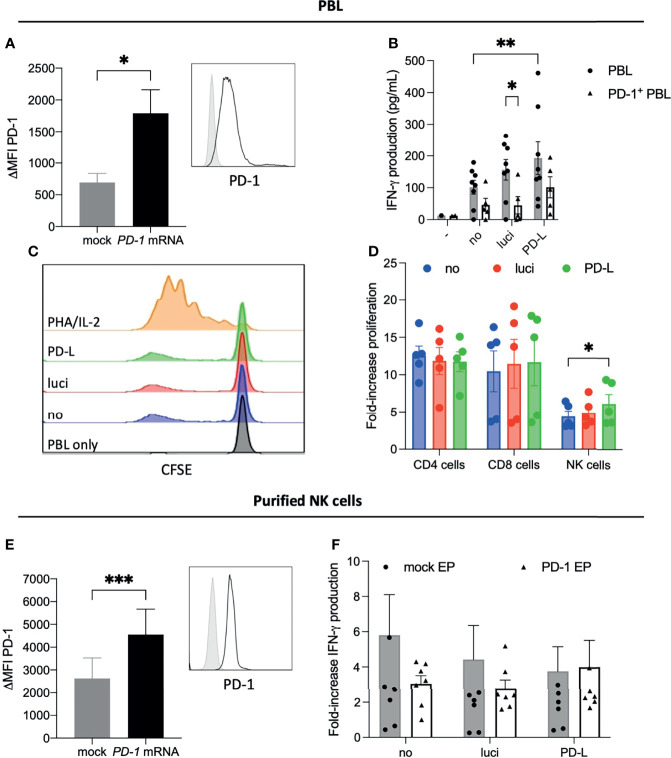
Stimulatory effect of siRNA engineered IL-15 DCs on primary human effector cells. **(A)** Mean fluorescence intensity of PD-1 surface expression on viable PBLs 2 hours after mock and *PD-1* mRNA electroporation (n = 3). Inset represents histogram overlay of PD-1 expression over isotype control (filled histogram). **(B–D)** Allo-stimulatory capacity of untreated (no), control siRNA (luci) or PD-L siRNA IL-15 DCs in a 5-day MLR with PBL (mock; circle symbols) and *PD-1* mRNA-electroporated PBL (PD-1^+^ PBL; triangle symbols). **(B)** Concentration IFN-γ in cell-free culture supernatant after 5 days stimulation (mean ± SEM; n = 9 in 4 independent experiments). **(C)** Degree of CFSE dilution in total viable PBL fraction is shown for one representative example of 6 donors, including a positive control with PHA/IL-2 and a negative control with unstimulated PBL. **(D)** Fold-increase of CFSE dilution upon stimulation with IL-15 DCs compared to their respective unstimulated mock-electroporated PBL, among singlet/Aqua live/dead^-^ gated CD3^+^CD4^+^ T cells, CD3^+^CD8^+^ and CD3^-^CD56^+^ NK cells (mean +- SEM, n = 6 in 3 independent experiments). **(E)** PD-1 expression on purified mock-electroporated or *PD-1* mRNA-electroporated NK cells (n = 8). Histogram shows a representative example of PD-1 expression in mock EP NK cells (grey filled histogram) and PD-1 EP NK cells (black unfilled histogram). **(F)** Fold-increase in concentration IFN-γ in 48h supernatants of cocultures of purified mock or *PD-1* mRNA-electroporated NK cells with autologous no, luci, or PD-L siRNA IL-15 DCs (n = 8). For **(A, E)** an unpaired t-test was used upon confirming normal distribution and homoscedasticity. For **(B, D)** the non-parametric Friedman test was used, *p < 0.05, ***p < 0.001. CFSE, carboxyfluorescein succinimidyl ester; EP, electroporation; IFN-γ, interferon gamma; MFI, mean fluorescence intensity; NK cell, natural killer cell; PBL, peripheral blood lymphocytes; PD-1, programmed death-1. **, 0.01.

Next, to investigate the impact of *in situ* PD-1 ligand disruption on the defining stimulatory function of DCs, allogeneic cell proliferation was assessed in a 5-day allo-MLR model using primary allo-PBL transfected with PD-1-encoding mRNA achieving overexpression of the inhibitory receptor. PD-1 expression was expressed on 85.25 ± 1.26% of viable PBL 2 hours after mRNA electroporation (ΔMFI of 1791.0 ± 165.7) and was significantly higher compared to its mock-transfected counterparts (ΔMFI 693.0 ± 102.0; **Figure 3A**, *p* = 0.0058, n=5). At the timepoint of analysis of supernatant and CFSE dilution, PD-1 expression after 5 days of coculture with IL-15 DCs, increased to 15.61 ± 1.08% for mock-transfected PBL and decreased for PD-1-electroporated PBL to 22.80 ± 2.52%.

Anticipating increased inhibitory signaling, PD-1-overexpressing PBL (black bars) released lower amounts of IFN-γ than the untransfected PBL (grey bars) upon stimulation with any of the IL-15 DCs, which was only significant in luci siRNA IL-15 DC stimulated PBLs (*p* = 0.027; **Figure 3B**). As a positive control, stimulation with PHA/IL-2 resulted in the highest secretion of IFN-γ exceeding the detection ranges, confirming functionality of PBL engineered to overexpress PD-1. PD-1^+^ PBL stimulated with control IL-15 DCs were capable of inducing IFN-γ (46.07 ± 20.38 pg/mL, no siRNA IL-15 DCs; 44.80 ± 27.06 pg/mL IFN-γ, luci siRNA IL-15 DCs), although not significantly higher than unstimulated PD-1^+^ PBL (11.97 ± 0.72 pg/mL IFN-γ), demonstrating the presence of inhibitory activity by introduced PD-1. Disrupting PD-1 ligand-mediated signaling by the DCs, PD-L siRNA IL-15 DCs triggered higher amounts of IFN-γ in PD-1-overexpressing PBL (101.33 ± 33.09 pg/mL) although not significant (**Figure 3B**). Interestingly, IFN-γ production by non-electroporated PBL was significantly higher when stimulated by PD-L siRNA IL-15 DCs (171.14 ± 75.46 pg/mL) compared to IL-15 DCs without siRNA treatment (68.32 ± 23.00 pg/mL, *p* = 0.022, n=5), but not by luci siRNA IL-15 DCs (141.31 ± 50.90).

Demonstrating favorable IFN-γ secretion by primary PBL stimulated by PD-L siRNA IL-15 DCs, we evaluated potential preferential expansion of PBL subsets by differently treated IL-15 DCs. DCs were co-cultured with CFSE-stained PBL and CFSE dilution after 5 days served as a measure for cell proliferation (**Figures 3C, D**). While all IL-15 DCs possess allo-stimulatory capacity, no significant increase in proliferation could be measured in CD4^+^ T cells and CD8^+^ T cells. Interestingly, NK cells stimulated with PD-L siRNA IL-15 DCs proliferated 6.10 ± 1.26 times more than unstimulated NK cells compared to 4.38 ± 0.71 times after stimulation with untreated IL-15 DCs (*p* = 0.048). Furthermore, we measured CD25 expression and CD69 expression, two activation markers on PBL at 1-day intervals during the allo-MLR coculture, but could not detect any difference between no, luci or PD-L siRNA treated IL-15 DCs at any of the timepoints (data not shown).

To further investigate the role of NK cells as effector cells in the immune response induced by IL-15 DCs and the relevance of the PD-1 signaling axis, autologous NK cells were electroporated with *PD-1* mRNA prior to coculturing with IL-15 DCs. Mock-electroporated NK cells showed a basal expression of PD-1 with a ΔMFI of 2619 ± 320.5 (**Figure 3E**), which increased to 4551 ± 396.4 after electroporation with PD-1 mRNA (*p* = 0.0006). In an autologous setting however, NK cells did not produce more IFN-γ in 24 hour and 48 hour co-cultures with PD-L siRNA IL-15 DCs (**Figure 3F**).

### PD-L1/2-Silenced IL-15 DCs Disrupt PD-1-Mediated Suppression of WT1-Specific T Cell Activity

**Figure 4:**
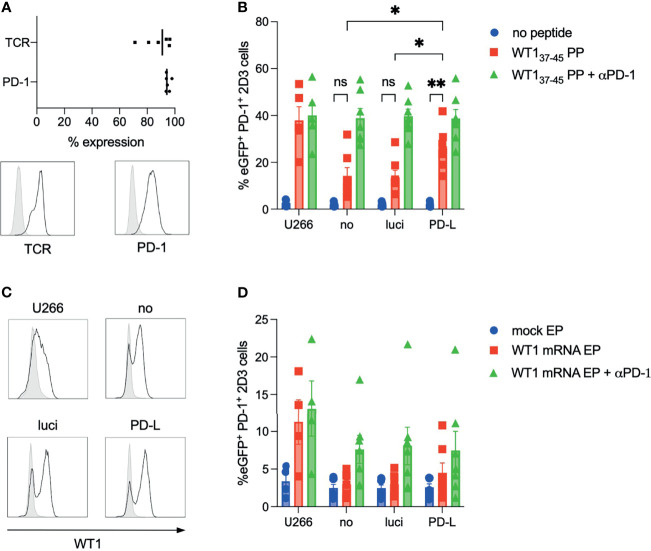
Improved WT1-specific T cell activation by silencing of PD-1 ligands in a model PD-1^+^ 2D3 T-cell model assay. **(A)** Percentage of PD-1 and TCRαβ surface expression in TCRαβ-deficient, PD-1-transduced and *WT1_37-45_ TCR* mRNA-electroporated 2D3 cells (n = 6, upper panel) and representative histogram overlays (lower panel) of TCRαβ and PD-1 staining (black line) with their respective isotype (grey filled line). **(B)** Percentage of eGFP-expressing 7-AAD^-^ CD8^+^ PD1^+^ 2D3 cells (n = 7) after overnight co-culture with untreated (no), control siRNA (luci) and PD-L siRNA (PD-L) IL-15 DCs that were either left unpulsed (no peptide) or pulsed with WT1_37-45_ peptide (PP). In a third condition, IL-15 DCs were peptide pulsed and neutralizing anti-PD-1 antibody (nivolumab) was added to the co-culture (WT1_37-45_ PP + αPD-1). **(C)** Representative histogram overlays of intracellular WT1 expression 4 hours after *WT1* mRNA electroporation (black line) versus mock (no mRNA) electroporation (grey filled line) of control APCs U266 cells and untreated (no), control siRNA (luci) and PD-L siRNA (PD-L) IL-15 DCs. **(D)** Percentage of eGFP-expressing 7-AAD^-^ CD8^+^ PD1^+^ 2D3 cells (n = 7) after co-culture with mock-electroporated or *WT1* mRNA-electroporated PD-L^-^ U266 cells or untreated (no), control siRNA (luci) or PD-L siRNA (PD-L) IL-15 DCs. Additionally, co-cultures with WT1 mRNA-electroporated APCs were pre-incubated with nivolumab. eGFP, enhanced green fluorescent protein; EP, electroporation; ns, not significant; PD-1, programmed death-1; TCR, T-cell receptor; WT1, Wilms’ tumor protein-1. *, 0.05; **, 0.01; NS, not significant.

Aiming to assess the added value of PD-L silencing in IL-15 DCs in an antigen-specific manner we previously developed a PD-1 overexpressing model T-cell line that can be readily transfected with antigen-specific TCR-encoding mRNAs of interest (20). In line with previous findings (20), maximal PD-1 surface expression (> 95%) was confirmed in these stably transduced PD-1^+^ 2D3 cells (**Figure 4A**). WT1_37-45_-specific *TCR* mRNA electroporation reproducibly resulted in a mean expression level of 87.72 ± 4.09% of TCRαβ^+^ PD-1^+^ 2D3 cells, 2 hours after transfection (**Figure 4A**). Next, we analyzed the expression of nuclear factor of activated T-cells (NFAT)-driven eGFP reporter gene by viable CD8^+^ PD-1^+^ 2D3 cells. Expression of eGFP, following co-culture with untreated, luci siRNA-treated and PD-L siRNA-treated IL-15 DCs that are exogenously pulsed with WT1_37-45_ peptide or left unpulsed, serves as a measure for WT1_37-45_-specific T cell activity (**Figure 4B**). WT1/PD-L1/PD-L2 triple-negative U266 cells were also exogenously pulsed with WT1_37-45_ peptide or left unpulsed, and were used as control APCs in this WT1-specific PD-1^+^ T-cell model assay (23). To determine whether reduced eGFP expression was caused by inhibitory signals mediated by PD-1/PD-L interaction, we included a condition in which WT1_37-45_ peptide and neutralizing anti-PD-1 antibody nivolumab were added to the co-cultures. Stimulation with U266 without antigen resulted in a mean background activation of 2.67 ± 0.60% eGFP^+^ PD-1^+^ 2D3 cells (n = 5). In the presence of specific antigen, PD-L^-^ U266 cells induced robust eGFP expression levels of 37.90 ± 5.82% and resulted in equally high T-cell activation when PD-1 was blocked by pre-incubation with the neutralizing antibody (40.02 ± 5.47% eGFP^+^ PD-1^+^ 2D3 cells), indicating that the maximal antigen-specific eGFP signal was reached (**Figure 4B**).

In line with control U266 cells, low background eGFP expressions were detected upon stimulation with untreated (no) IL-15 DCs (1.94 ± 0.32%), luci siRNA IL-15 DCs (2.03 ± 0.31%) and PD-L siRNA IL-15 DCs (2.21 ± 0.36%; for 7 independent donors) in the absence of peptide. Co-culture of TCR-engineered PD-1^+^ 2D3 cells with non-silenced IL-15 DCs, which showed an average expression of 97.57 ± 2.13% PD-L1 and 9.80 ± 4.78% PD-L2 (7 independent donors) and that were pulsed with the corresponding WT1_37-45_ peptide, resulted in low eGFP expression (14.24 ± 3.56%), similar to coculture with luci siRNA IL-15 DCs (13.64 ± 2.94% eGFP^+^ PD-1^+^ 2D3 cells). eGFP levels were not significantly different in these two conditions from the negative control without peptide. However, blocking of PD-1/PD-L interaction with nivolumab disrupted the suppressive effect, resulting in maximal eGFP expression by WT1_37-45_-specific TCR-engineered PD-1^+^ 2D3 cells when co-cultured with peptide-pulsed no siRNA IL-15 DCs (38.81 ± 4.13%) and luci siRNA IL-15 DCs (39.66 ± 3.08% eGFP^+^ PD-1^+^ 2D3 cells). These results confirm that the nearly absent T-cell activity is caused by active PD-1-mediated inhibition. Proving the concept of enhancing the antigen-specific T cell-stimulating capacity of IL-15 DCs by *in situ* silencing of PD-1 ligands, WT1_37-45_ peptide-loaded PD-L-silenced IL-15 DCs – with mean relative expression of 16.58 ± 2.05% PD-L1 and 27.91 ± 7.70% PD-L2 – induced significantly higher specific T cell activation (26.04 ± 3.60% eGFP^+^ PD-1^+^ 2D3 cells) than their non-silenced counterparts (*p* = 0.035 compared to no siRNA IL-15 DCs and *p* = 0.024 compared to luci siRNA IL-15 DCs), approximating the maximal activation potential in the presence of neutralizing anti-PD-1 antibody (38.67 ± 3.88% eGFP^+^ PD-1^+^ 2D3 cells).

We further exploited the possibilities of this PD-1^+^ 2D3 based T-cell assay, by evaluating the tumor antigen-presenting capacity of PD-L-disrupted IL-15 DCs loaded with mRNA encoding the full-length WT1 protein. Contrary to exogenous peptide pulsing, transfection of DCs with *WT1* mRNA allows, in principle, endogenous processing and presentation of the full WT1 epitope repertoire. *WT1* mRNA transfection efficiency was demonstrated by intracellular WT1 protein expression with flow cytometry (**Figure 4C**). Mean expression levels of intracellular WT1 4 hours after *WT1* mRNA electroporation were comparable for all conditions, with 35.97 ± 11.14% WT1^+^ U266 cells (n=4), 47.31 ± 10.23% WT1^+^ siRNA untreated (no) IL-15 DCs, 53.27 ± 11.03% WT1^+^ luci IL-15 DCs and 47.75 ± 12.51% WT1^+^ PD-L siRNA IL-15 DCs (n=7; **Figure 4C**, representative example). To assess whether *WT1* mRNA-loaded and PD-L silenced IL-15 DCs can efficiently stimulate TCR-engineered PD-1^+^ 2D3 cells in an antigen-specific manner, co-cultures of WT1_37-45_ TCR-engineered PD-1 and the different IL-15 DCs conditions were set up similar to those used with WT1_37-45_ peptide pulsing (**Figure 4D**). In line with non-peptide pulsed control U266 cells, mock-electroporated (no *WT1* mRNA) U266 cells triggered low background percentages eGFP expression (3.37 ± 0.99% eGFP^+^ PD-1^+^ 2D3 cells). However, endogenously processing of WT1 in PD-L^-^ U266 cells after *WT1* mRNA electroporation triggered a 3-fold increase in eGFP expression compared to the mock-electroporated control (11.28 ± 3.00% eGFP^+^ PD-1^+^ 2D3, 4 independent replicates), although this difference is not statistically significant. Elevated eGFP expression induced by *WT1* mRNA-electroporated U266 cells suggests that WT1_37-45_ peptide is indeed processed and presented on the surface of U266 cells, as also reported in (23). As observed with WT1_37-45_ peptide-pulsed U266 cells, blocking PD-1 inhibitory signal with anti-PD-1 antibody on the PD-1^+^ 2D3 cells had no impact on eGFP expression (13.07 ± 3.72% eGFP^+^ PD-1^+^ 2D3), since U266 cells do not express PD-1 ligands. Background T-cell activation by any of the mock-electroporated IL-15 DCs conditions was reproducibly low with average percentages of eGFP^+^ PD-1^+^ 2D3 cells of 2.49 ± 0.47% for no siRNA, 2.46 ± 0.44% for luci siRNA and 2.58 ± 0.45% for PD-L siRNA conditions (n=7). WT1_37-45_ epitope presentation by *WT1* mRNA-electroporated IL-15 DCs induced 2.98 ± 0.59% (no siRNA) and 3.01 ± 0.53% (luci siRNA) eGFP^+^ PD-1^+^ 2D3 cells (n=7), therefore, not surpassing background activation levels. However, by blocking PD-1 signaling with nivolumab, WT1_37-45_ specific activation by these untreated (no siRNA) *WT1* mRNA-loaded IL-15 DCs (7.62 ± 1.86%) and control siRNA (luci) *WT1* mRNA-loaded IL-15 DCs (8.08 ± 2.49%), was robustly 2,5-fold higher than the respective mock conditions (n=7), albeit not statistically significant. On the contrary, *WT1* mRNA-electroporated PD-L siRNA IL-15 DCs were capable of triggering above-background eGFP expression levels in PD-1^+^ 2D3 cells in the absence of nivolumab (4.51 ± 1.31% eGFP^+^ PD-1^+^ 2D3 cells), compared to mock-electroporated PD-L siRNA IL-15 DCs although not significant. In the presence of nivolumab, PD-L siRNA IL-15 DCs were able to stimulate even more PD-1^+^ 2D3 cells (7.47 ± 2.56% eGFP^+^ PD-1^+^ 2D3 cells) to the same level as no siRNA or luci siRNA IL-15 DCs.

## Discussion

Although DC vaccination has proven to be effective in a number of cancer patients, the lack of durable and widespread responses urges the improvement of their immunogenicity through further research. In this paper, we developed a new DC vaccine candidate and evaluated it for its immunostimulatory properties. The discovery of the role of PD-1 in dampening anti-tumor immunity and antibodies targeting this inhibitory immune checkpoint or its ligands have revolutionized the field of cancer immunotherapy (24). This finding has led to PD-1/PD-L1 blocking antibody therapy being among the most often applied and promising cancer treatments (25). Increasing insights in the effects of systemic PD-1 checkpoint blockade has brought forward that this type of therapy is generally well tolerated, but can often result in several immune-related adverse effects, some of which have a severe nature (26). To increase the safety potential of PD-1 checkpoint blockade we hypothesized that neutralizing PD-1 ligands in a DC vaccine could increase the strength of the vaccine, while avoiding severe adverse effects by omitting systemic anti-PD-1 or anti-PD-L therapy. Notably, during the priming phase of T cells, which determines the fate of naïve T cells, the balance between co-stimulation and co-inhibition plays a major role. By decreasing the expression of co-inhibitory molecules, the T-cell fate is more likely to shift towards activation and induction of immunological memory, which is highly favorable as many TAAs are in fact self-antigens and thereby poor inducers of T cell responses (27).

In conventional IL-4 DCs, silencing PD-1 ligands resulted in increased IFN-γ production by allogeneic and antigen-specific T cells, both *ex vivo* and *in vivo* (17, 18). Importantly, combination with IL-15 transpresentation further augmented these responses (19). Our lab has previously generated a novel DC vaccine with improved immunostimulatory properties compared to conventional IL-4 DCs, so called-IL-15 DCs (7). In this study, we evaluated whether PD-L silencing could also further improve the immunopotency of the IL-15 DCs. Incorporation of PD-L siRNAs in the IL-15 DC protocol required alterations in the silencing protocol that was previously used in IL-4 DCs. PD-L siRNA transfection in IL-4 DCs was done by harvesting immature IL-4 DCs at day 3 and subsequent transfection with siRNAs followed by an extra differentiation step of 4 days and a maturation step of 2 days (16–19). However, the 3-day culture protocol of IL-15 DCs makes this siRNA transfection strategy unsuitable. Furthermore, as PD-L1 and PD-L2 expression is upregulated during the differentiation and maturation from monocytes into IL-15 DCs, delivery of siRNAs at the beginning of the culture (i.e. before proteins are expressed by the cells) would be most favorable. Therefore, we opted for delivery of siRNAs at the monocyte stage of the culture. However, this resulted in low yield and viability, possibly due to the more fragile nature of monocytes compared to immature DCs. Upon further optimizations we established a protocol where PD-1 ligands were efficiently silenced below a relative threshold value of 50% PD-L expression. This cut-off value was according to the criteria of the ongoing clinical trial using PD-L silenced IL-4 DCs by Dr. Schaap (NCT02528682, clinicaltrials.gov). An absolute knock-out of PD-L1 and PD-L2 was not considered favorable. First, expression of co-inhibitory molecules on the DCs can protect them from cytotoxic T-cell-mediated killing, prolonging their persistence and function. Second, although PD-L2 is usually seen as a co-inhibitory molecule, it is also demonstrated to have costimulatory properties, possibly *via* its interaction with repulsive guidance molecule B (28–30). Finally, a certain degree of co-inhibition is still desirable as overstimulation of immune cells can result in activation-induced cell death (31).

The role of effector T cells in the anti-tumor immune response is long-known and established, as well as PD-1 interactions in this context. On the contrary, involvement of NK cells and their PD-1 expression status in the immune response is only substantiated during the last decades, as is the bidirectional crosstalk between DCs and NK cells (32–37). In this regard, a prominent role of IL-15 is described, either membrane bound (9) or transpresented *via* IL-15 receptor α (38). Recently, many reports document on the expression of PD-1 observed in NK cells in relation to their functional exhaustion, both in cancer patients (39–41) and in patients with chronic infections (42). Given the crosstalk between NK cells and DCs on the one hand, and expression of PD-1 on exhausted NK cells and PD-Ls on DCs, on the other hand, there might be a rationale for PD-L silencing on DCs to also reinvigorate NK-cell functions. To robustly guarantee PD-1 expression on PBLs, we used *PD-1* mRNA electroporation to allow for pairwise analyses between unmanipulated and PD-1^+^ PBLs (43). In line with our hypothesis, we demonstrated that PD-L siRNA IL-15 DCs increase cytokine production in an allo-MLR reaction. Because this effect was also present in luci siRNA IL-15 DCs, albeit at a less pronounced level, the contribution of the transfection reagent could not be excluded. Indeed, it has been shown, although not for SAINT-RED, that certain transfection reagents can result in differential gene expression due to their foreign nature as seen by the cell (44). However, as the effect was variable and still modest, we believe that the main effect in these set of experiments is due to the introduced siRNA and not the transfection reagent, although a synergistic function cannot be excluded. To our surprise, the stimulatory effect of PD-L silenced DCs was more pronounced in allogeneic PBLs that did not overexpress PD-1 compared to *PD-1* mRNA transfected PBLs, indicating such high inhibitory activity of the overexpressed PD-1, that it could not be overcome by PD-L silenced DCs. Indeed, PD-1^high^ T cells have distinct gene expression profiles compared to PD-1^low^ and PD-1^intermediate^ T cells, with the first expressing high levels of genes related to exhaustion (45). Although we did not induce PD-1 expression in a physiological manner, thereby generating a *bona fide* exhaustion model, the mere introduction of PD-1 was sufficient to inhibit IFN- γ secretion by PD-1^+^ PBLs. Attempting to ascribe this IFN- γ production to a certain cell type, a proliferation assay identified NK cells, but not T cells to be expanded after stimulation with PD-L siRNA IL-15 DCs. Further focusing on NK cells, no difference could be seen in IFN-γ production by NK cells between no siRNA IL-15 DCs and PD-L siRNA IL-15 DCs in an autologous setting. It is likely that absence of killer immunoglobulin-like receptor ligand mismatch in an autologous setting limits the IFN-γ production by NK cells (46). The absence of a significantly increased proliferative response in the T-cell compartment by PD-L downregulated DCs is in accordance with previous findings by others (47). Because the true potential of DC vaccination on T-cell activation relies on their ability to stimulate antigen-specific T cells, we focused on the WT1_37-45_ epitope for the remainder of the paper. In an in-house developed assay to assess the PD-1^+^ T-cell stimulating capacity of APCs, PD-L silenced IL-15 DCs showed stronger T-cell stimulatory capacities compared to IL-15 DCs with naturally expressed PD-1 ligands. Robustly demonstrated when DCs were loaded with one specific epitope, PD-L silenced DCs also outperformed their control counterparts when loaded with WT1-encoding mRNA. The lower response in this setting can be explained by the fact that the 2D3 cells in this assay only recognize one specific epitope which is the exact epitope used in the peptide-pulsed conditions, while mRNA loading results in multi-epitope presentation. Towards clinical implementation, mRNA loading is of great interest as this thus can result in a multi-epitope immune response by both CD4^+^ and CD8^+^ T cells (48). In this way other cancer-related epitopes might be presented that are not yet known. Moreover, this strategy requires no prior knowledge about the HLA-haplotype of patients (49), while the peptide pulsing approach requires the expression of correct HLA molecules.

The data in this paper demonstrate that PD-L siRNA IL-15 DCs are capable of stimulating tumor-antigen T cells, even when PD-1 is overexpressed. In this way, PD-L siRNA IL-15 DCs might reinvigorate exhausted tumor-reactive CD8^+^ T cells. In the context of tackling inhibitory mechanisms with DC vaccination, regulatory T cells (Tregs) are gaining interest regarding the immune suppressive tumor micro-environment and their significance as a therapeutic target in cancer (50–53). It has been reported that PD-L1-mediated interactions with naïve CD4^+^ T cells play an important role in the development, maintenance and function of inducible Tregs (54). Using nanoparticles coated with or without PD-L1, Francisco and colleagues demonstrated that in the absence of PD-L1 the development of inducible Tregs was reduced (54). Thus, PD-L siRNA IL-15 DCs might also be beneficial in the generation of a less immune suppressive tumor micro-environment. Further research is warranted to fully delineate the added value of PD-L siRNA IL-15 DCs as a potential new anticancer vaccine.

## Conclusion

The PD-1/PD-L checkpoint axis is an important mediator of exhaustion in several immune effector cells in cancer. To advance next-generation DC vaccines with increased immunopotency, we successfully developed a robust protocol incorporating disruption of PD-1 ligands in our latest short-term cultured IL-15 DC vaccine, preserving all prototypic DC phenotype and functional characteristics. Ultimately designed to induce a durable tumor-specific immune response, PD-L silenced IL-15 DCs were capable of rescuing antigen-specific cytotoxic T cells from PD-1-mediated inhibition. Further corroborating the superior potency of short-term IL-15 DCs, the combination of immune stimulatory components during DC differentiation and maturation with *in situ* checkpoint inhibition supports further clinical translation.

## Data Availability Statement

The raw data supporting the conclusions of this article will be made available by the authors, without undue reservation.

## Ethics Statement

The studies involving human participants were reviewed and approved by Comité voor Medische Ethiek, Universiteit Antwerpen. The patients/participants provided their written informed consent to participate in this study.

## Author Contributions

MV and EL conceptualized the research. MV, DF, HR, LB, and SP performed the experiments. MV and EL wrote the manuscript. MV, DF, DC-D, HR, LB, SP, WH, ES, and EL critically reviewed and edited the manuscript. All authors read and approved the manuscript.

## Funding

The research in this manuscript was supported by a “Cellular Immunotherapy” grant from the vzw Baillet Latour Fund (Belgium), the Cellular Therapy Fund from the Antwerp University Hospital (UZA) Foundation, the Foundation Against Cancer (Stichting tegen Kanker, Belgium), Stand up against Cancer (Kom Op Tegen Kanker, Belgium) and grant FFB160025 from the Special Research Fund (BOF, University of Antwerp, Belgium). MV was supported by grant 1S24517N from the Research Foundation Flanders (FWO, Belgium). DC-D was supported by a DOC-PRO PhD grant from the BOF and by grant G053518N from the FWO.

## Conflict of Interest

The authors declare that the research was conducted in the absence of any commercial or financial relationships that could be construed as a potential conflict of interest.

## Publisher’s Note

All claims expressed in this article are solely those of the authors and do not necessarily represent those of their affiliated organizations, or those of the publisher, the editors and the reviewers. Any product that may be evaluated in this article, or claim that may be made by its manufacturer, is not guaranteed or endorsed by the publisher.

## Supplementary Material

Supplementary Figure 1 Schematic overview of the PD-L siRNA monocyte-derived 3-day IL-15 DC culture protocol. After a 1-hour incubation of monocytes with lipoplexes of SAINT-RED (10 µL) with a mixture of PD-L1:PD-L2 siRNAs (2 µg siRNAs; 2:1 ratio), cells were differentiated for 24 to 48 hours and matured overnight into PD-L siRNA IL-15 DCs, without intermediate harvesting. DC, dendritic cell; GM-CSF, granulocyte macrophage colony-stimulating factor; hAB, human pooled serum; iDC, immature dendritic cell; IFN, interferon; IL, interleukin; PD-L, programmed death-1 ligands; PD-L1, programmed death-1 ligand 1; PD-L2, programmed death-1 ligand 2; PGE, prostaglandin; siRNA, small interfering ribonucleic acid; TNF, tumor necrosis factor. **Supplementary Figure 1** was created with BioRender.

Supplementary Figure 2 Gating strategy employed in the allo-MLR experiment. First cells were gated based on FSC/SSC and viability. Then CD3^+^ cells were gated to select CD4^+^ and CD8^+^ cells downstream. CD3^+^CD4^+^ and CD3^+^CD8^+^ cells were selected to study proliferation based on CFSE dilution. CFSE-dilution gate was set on unstimulated PBL.

Supplementary Figure 3 Gating strategy used in 2D3 assay. First 2D3 cells were gated based on their scatter profile. Then viable cells were selected by means of 7-AAD. CD8^+^ cells were then selected, depicting the CD8^+^ 2D3 cells. Finally, T cell activation is measured on these cells by measuring eGFP expression. The eGFP^+^ gate was set on unstimulated 2D3 cells.
