# Supplementary figures and images for: Anti-Tumor Potency of Short-Term Interleukin-15 Dendritic Cells Is Potentiated by In Situ Silencing of Programmed-Death Ligands
Source: Front Immunol. 2022 Feb 17;13:734256. doi: 10.3389/fimmu.2022.734256 (PMC8891487; doi:10.3389/fimmu.2022.734256)

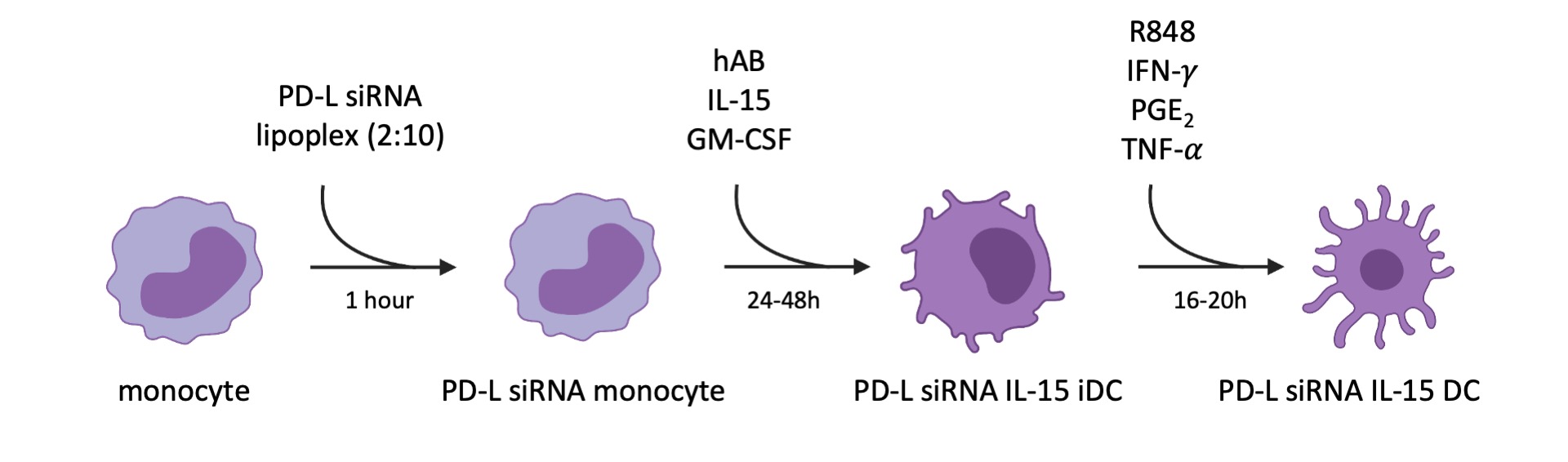

Supplement: Supplementary Figure 1 — Schematic overview of the PD-L siRNA monocyte-derived 3-day IL-15 DC culture protocol. After a 1-hour incubation of monocytes with lipoplexes of SAINT-RED (10 µL) with a mixture of PD-L1:PD-L2 siRNAs (2 µg siRNAs; 2:1 ratio), cells were differentiated for 24 to 48 hours and matured overnight into PD-L siRNA IL-15 DCs, without intermediate harvesting. DC, dendritic cell; GM-CSF, granulocyte macrophage colony-stimulating factor; hAB, human pooled serum; iDC, immature dendritic cell; IFN, interferon; IL, interleukin; PD-L, programmed death-1 ligands; PD-L1, programmed death-1 ligand 1; PD-L2, programmed death-1 ligand 2; PGE, prostaglandin; siRNA, small interfering ribonucleic acid; TNF, tumor necrosis factor. Supplementary Figure 1 was created with BioRender. [file Image_1.jpeg]

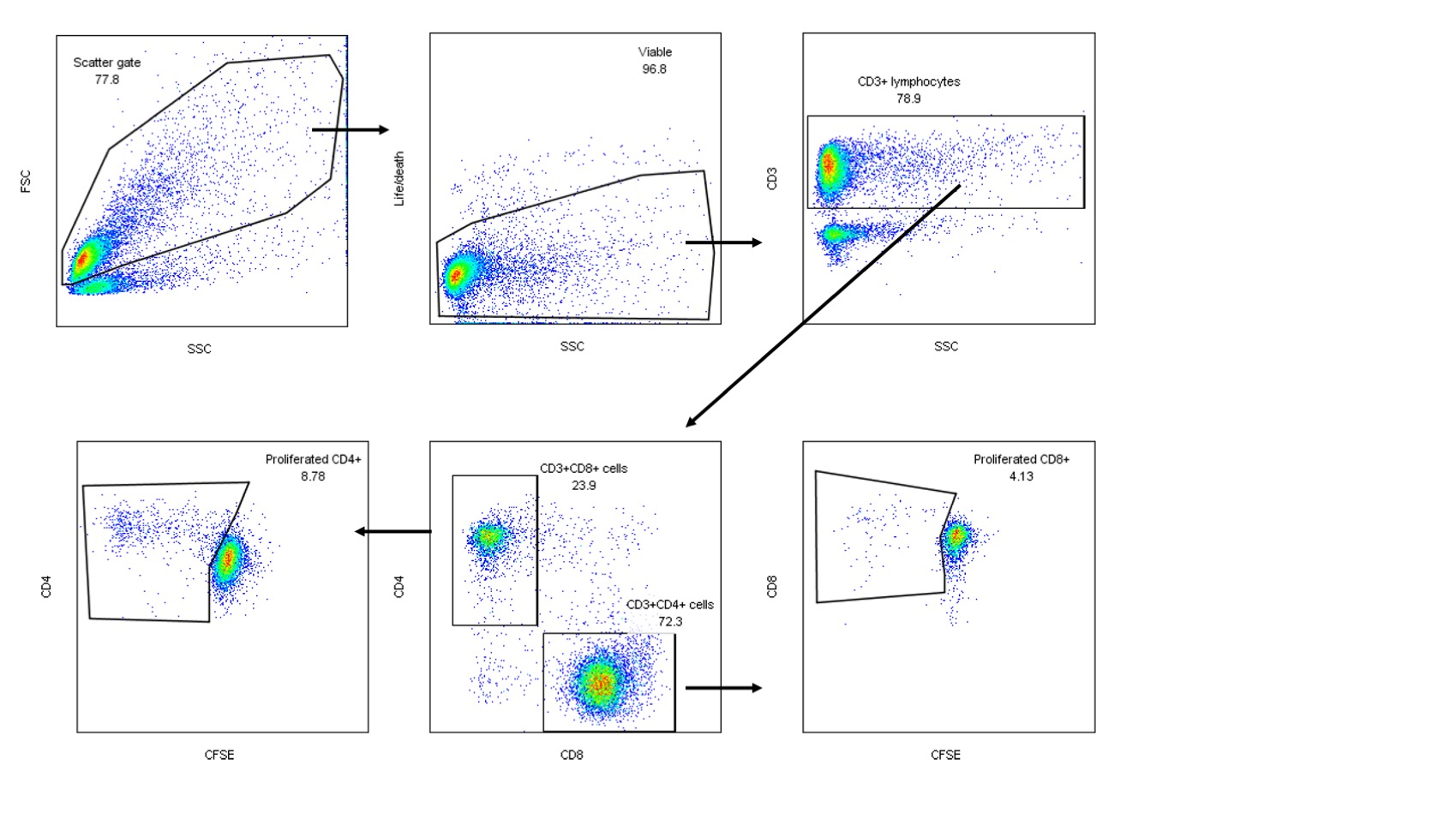

Supplement: Supplementary Figure 2 — Gating strategy employed in the allo-MLR experiment. First cells were gated based on FSC/SSC and viability. Then CD3+ cells were gated to select CD4+ and CD8+ cells downstream. CD3+CD4+ and CD3+CD8+ cells were selected to study proliferation based on CFSE dilution. CFSE-dilution gate was set on unstimulated PBL. [file Image_2.jpeg]

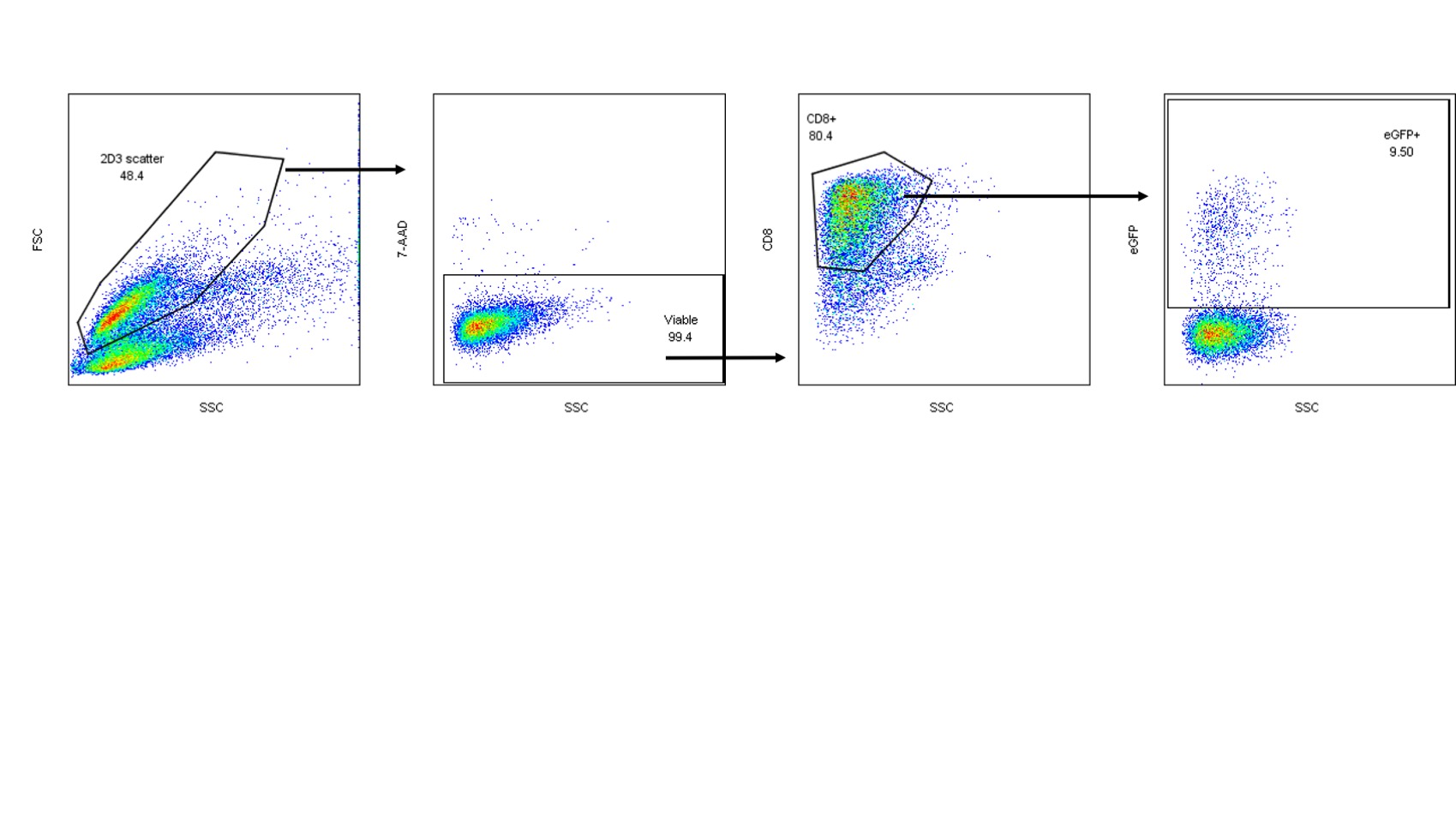

Supplement: Supplementary Figure 3 — Gating strategy used in 2D3 assay. First 2D3 cells were gated based on their scatter profile. Then viable cells were selected by means of 7-AAD. CD8+ cells were then selected, depicting the CD8+ 2D3 cells. Finally, T cell activation is measured on these cells by measuring eGFP expression. The eGFP+ gate was set on unstimulated 2D3 cells. [file Image_3.jpeg]
